# Three Diseases Mediated by Different Immunopathologic Mechanisms—ANCA-Associated Vasculitis, Anti-Glomerular Basement Membrane Disease, and Immune Complex-Mediated Glomerulonephritis—A Common Clinical and Histopathologic Picture: Rapidly Progressive Crescentic Glomerulonephritis

**DOI:** 10.3390/biomedicines11112978

**Published:** 2023-11-06

**Authors:** Cristina Gluhovschi, Florica Gadalean, Silvia Velciov, Mirabela Nistor, Ligia Petrica

**Affiliations:** 1 Division of Nephrology, “Victor Babeș” University of Medicine and Pharmacy, 300041 Timișoara, Romania; fnnicorici@yahoo.com (F.G.); ligia_petrica@yahoo.co.uk (L.P.); 2 Centre for Molecular Research in Nephrology and Vascular Disease, Faculty of Medicine, “Victor Babeș” University of Medicine and Pharmacy, Eftimie Murgu Sq. No. 2, 300041 Timișoara, Romania; s_velciov@yahoo.com; 3 Division of Nephrology, County Emergency Hospital Timisoara, 300041 Timișoara, Romania

**Keywords:** rapidly progressive crescentic glomerulonephritis, ANCA-associated vasculitis, anti-GBM antibodies, immune complex-mediated glomerulonephritis

## Abstract

Immune mechanisms play an important role in the pathogenesis of glomerulonephritis (GN), with autoimmunity being the main underlying pathogenetic process of both primary and secondary GN. We present three autoimmune diseases mediated by different autoimmune mechanisms: glomerulonephritis in vasculitis mediated by anti-neutrophil cytoplasmic antibodies (ANCAs), glomerulonephritis mediated by anti-glomerular basement membrane antibodies (anti-GBM antibodies), and immune complex-mediated glomerulonephritis. Some of these diseases represent a common clinical and histopathologic scenario, namely rapidly progressive crescentic glomerulonephritis. This is a severe illness requiring complex therapy, with the main role being played by therapy aimed at targeting immune mechanisms. In the absence of immune therapy, the crescents, the characteristic histopathologic lesions of this common presentation, progress toward fibrosis, which is accompanied by end-stage renal disease (ESRD). The fact that three diseases mediated by different immunopathologic mechanisms have a common clinical and histopathologic picture reveals the complexity of the relationship between immunopathologic mechanisms and their clinical expression. Whereas most glomerular diseases progress by a slow process of sclerosis and fibrosis, the glomerular diseases accompanied by glomerular crescent formation can progress, if untreated, in a couple of months into whole-nephron glomerulosclerosis and fibrosis. The outcome of different immune processes in a common clinical and histopathologic phenotype reveals the complexity of the relationship of the kidney with the immune system. The aim of this review is to present different immune processes that lead to a common clinical and histopathologic phenotype, such as rapidly progressive crescentic glomerulonephritis.

## 1. Introduction

Immunopathology is characterized by diverse immune mechanisms. These can be limited or systemic. They can affect a single organ—the kidneys, for example, in primary glomerulonephritis (GN)—or two organs, the kidneys and the lungs, such as in Goodpasture’s syndrome. In other situations, immune mechanisms are systemic, affecting several organs. For example, in systemic lupus erythematosus (SLE), immune mechanisms target the kidneys, skin, serous membranes (pericardium, joints, and pleura), and brain, producing a severe illness with protean clinical manifestations.

Many glomerulonephritides are autoimmune-mediated. Glomerulonephritis can be a component of an autoimmune disorder affecting multiple organs, such as in SLE. Renal involvement, namely lupus nephritis, can be the dominant clinical phenotype. Other autoimmune diseases are limited to a single organ, such as autoimmune thyroiditis or renal limited vasculitis.

The clinical and histopathologic picture of an immune-mediated disease usually reflects the specific underlying immune mechanisms involved.

For example, in IgA nephropathy, immune complexes containing IgA antibodies appear in immune deposits at the kidney level, which clinically manifests as GN; however, histopathologically, this manifests as proliferative GN.

In a previous study, we suggested a possible relationship between immune and clinical manifestations in Rowell’s syndrome, which is a particular form of SLE associated with specific cutaneous manifestations. Its biological features present SLE characteristics and elements indicating the specificity of this syndrome (speckled ANA, anti-Ro antibodies, and positive rheumatoid factor or anti-La antibodies) [1].

Rapidly progressive glomerulonephritis (RPGN) represents another form of immune process manifestation. Three autoimmune diseases with totally different pathogenetic mechanisms cause similar renal lesions, namely crescent formation at the glomerular level.

These autoimmune diseases are represented by anti-neutrophil cytoplasmic antibody (ANCA)-associated vasculitis, glomerulonephritis mediated by anti-glomerular basement membrane antibodies (anti-GBM antibodies), and glomerulonephritis mediated by immune complexes, whose main representative is SLE.

All of these mechanisms produce a common clinical picture, which is defined as RPGN. This is a disease that presents with acute nephritic syndrome (including edema, proteinuria, hematuria, and hypertension) accompanied by severe renal functional impairment, which takes a rapidly progressive course (days, weeks, months) toward end-stage renal disease (ESRD) that can be lethal without specific therapy. The marked reduction in glomerular filtration rate usually limits the rate of protein filtration; the nephrotic syndrome is unusual and is most likely to occur in patients with less severe kidney function impairment.

Renal histopathologic examination reveals extensive crescent formation (in ≥50% of the glomeruli). Jennette considered that glomerular crescent formation seems to represent a non-specific response to a severe lesion of the glomerular capillary wall. Crescentic glomerulonephritis should represent the final common pathway in which the three autoimmune diseases are expressed [2].

It should be mentioned that therapies targeting the three autoimmune diseases under discussion are similar, including corticosteroids, immunosuppressive or immunomodulating drugs, and plasma exchange.

Until the etiologic agents of these diseases and their specific treatments are discovered, therapies targeting their underlying immune mechanisms remain the solution.

The aim of this paper is to present the clinical, biological, and histopathologic elements specific for each of these diseases and to discuss the relationship between their underlying immune mechanisms and the capillary wall lesions resulting in crescent formation at the glomerular level.

Finally, we present crescentic glomerulonephritis as a manifestation that is common to all three autoimmune diseases, synthesizing the ways in which this severe disease progressively evolves toward ESRD.

## 2. ANCA-Associated Vasculitis

Glomerulonephritis in ANCA-mediated vasculitis represents the most common cause of crescentic glomerulonephritis. This group, which is defined as ANCA-associated vasculitis according to the Chapel Hill Consensus Conference Classification 2012, includes granulomatosis with polyangiitis (GPA, formerly Wegener’s granulomatosis), microscopic polyangiitis (MPA), renal limited vasculitis (RLV), idiopathic RPGN, and eosinophilic granulomatosis with polyangiitis (EGPA, also defined as Churg-Strauss syndrome) [3].

Epidemiological studies carried out in Great Britain over a period of 10 years showed an overall annual incidence of primary systemic vasculitis of 19.8/million [4].

ANCA-associated vasculitis is frequently accompanied by renal involvement in the form of glomerulonephritis. The clinical picture is usually represented by the nephritic syndrome. The underlying histopathologic picture includes rapidly progressive crescentic glomerulonephritis.

The central feature is pauci-immune focal segmental necrotizing glomerulonephritis that may become crescentic with the accumulation of macrophages and epithelial cells in Bowman’s space. Histologically, ANCA-associated vasculitis is a necrotizing vasculitis with few or no immune deposits that predominantly affects small vessels. Necrotizing glomerulonephritis is less common in EGPA, particularly ANCA-negative EGPA [5]. Moderate to marked vessel wall immune deposition is a characteristic of immune complex-mediated small vessel vasculitis [6]. GPA, MPA, and RLV severely impair renal vasculature. EPGA mainly manifests at the respiratory tract level.

ANCA antibodies are involved in the pathogenesis of ANCA-associated vasculitis. They act on neutrophils. ANCA antibodies recognizing perinuclear targets are defined as p-ANCA, with the main ones being directed at myeloperoxidase (MPO). ANCA antibodies targeting antigens located in the cytoplasm are defined as cytoplasmic ANCA or c-ANCA and are mainly directed at proteinase 3 (PR3).

Activated neutrophils release proteases, which, in combination with reactive oxygen species and complement system components, react with the vascular endothelium to produce lesions [7]. Gaps in the capillary wall are produced, through which fibrin, macrophages, as well as T and B cells, pass from the capillary wall into the extra-capillary space [8].

When they reach this level, they interact with cells bordering this area, namely the parietal epithelial cells of Bowman’s capsule, as well as podocytes. These cells react, detach, and penetrate the filtration area, which is inefficient in removing the new elements that are present. The cells group themselves and take the form of crescents in which epithelial cells are initially predominant. Around the glomerulus, an interstitial inflammatory reaction occurs that is composed of cells such as monocytes/macrophages, T cells, and fibroblasts. Macrophages can disrupt Bowman’s capsule and penetrate the filtration area, which worsens the phenomena at this level [9]. Crescents contain not only epithelial cells (epithelial crescents) but also monocyte/macrophages, which form more complex cellular crescents that attract T cells.

ANCA antibodies also act on small, pre-glomerular vessels to produce inflammatory lesions at the endothelium level [10]. Coagulation abnormalities are accompanied by impaired fibrinolysis because of the action of anti-fibrinolytic antibodies. These lead to thrombi formation while the capillary wall undergoes fibrinoid necrosis [11].

Small vessel impairment can also occur in the lungs, which involves cell extravasation (including red cells with ensuing pulmonary hemorrhage) from the blood into alveoli and ensuing respiratory failure. Pulmonary phenomena, as well as allergic phenomena, are the mainstay in EPGA; however, renal phenomena are less evident or absent.

In renal-limited vasculitis, respiratory phenomena are absent. In these cases, the pathologic process is limited to the kidney.

In ANCA-associated vasculitis, especially in GPA, granulomas can form in the kidneys. They are usually located at the interstitial level. GPA granulomas can also appear in other organs, for example, in the lungs [12].

Regarding immune complex and complement deposits, it should be mentioned that these deposits are either absent or present in very limited quantities, hence the name pauci-immune GN. Immune complexes, when present in the glomeruli, are considered to be the result of passive trapping of circulating immune complexes, and their pathogenetic role is controversial. Recent research, however, has suggested that the complement system, especially the alternate pathway, has an important role in the pathogenesis of ANCA-associated vasculitis [11,13]. Not all glomeruli are affected in ANCA-associated vasculitis, as glomeruli with crescents occur at variable proportions. Rapidly progressive crescentic glomerulonephritis is thought to be present in at least 50% glomeruli involved, and the percentage of the involved glomeruli can dictate the disease outcome.

If there is no other underlying kidney disease, the unaffected glomeruli and non-necrotic lesions in segmentally affected glomeruli look unremarkable. Less commonly, patients may show extraglomerular renal vasculitis.

ANCA-associated GN can occur in four forms, as noted in research studies of the European Vasculitis Study Group (EUVAS), including focal, crescentic, mixed, and sclerotic forms [14].

GPA is mainly associated with c-ANCA, and MPA is mainly associated with p-ANCA.

Some ANCA antibodies can also be found in normal people, however in low concentrations [15]. ANCA antibodies act not only on neutrophils but also on monocytes [16].

The pathogenetic role of ANCAs has been demonstrated in both experimental and human studies. The pathogenetic potency of ANCAs seems to be related to epitope specificity [17].

Both in vivo and in vitro studies have shown that anti-MPO-ANCAs can induce necrotizing small vessel vasculitis and glomerulonephritis, suggesting an important pathogenetic role. Granuloma formation, however, in anti-PR3-ANCA-associated GPA is not well explained by the presence of autoantibodies in experimental models [18]. It should be mentioned that myeloperoxidase is not the only p-ANCA antigen. In recent years, a novel ANCA antigen, lysosome-associated membrane protein-2 (LAMP-2), has been described [19]. In ANCA-associated vasculitis, other antibodies have been described as well, including anti-plasminogen and anti-moesin antibodies [20].

LAMP-2 antibodies represent a sub-type of ANCA autoantibodies. They can interact directly with the endothelium to produce lesions at this level. LAMP-2 antibodies can also act on neutrophils and activate them [21]. It is important to mention that an epitope of human lysosome-associated membrane protein 2 (hLAMP-2) is homologous with a bacterial adhesion molecule [22]. The LAMP-2 epitope recognized by ANCAs mimics microbial antigens [23].

Infectious factors acting as triggers can also contribute to vasculitis pathogenesis [24]. In granulomatosis with polyangiitis (GPA), this role is attributed to Staphylococcus aureus. A prophylactic role for trimethoprim sulfamethoxazole therapy has been shown [25].

The PR3 antigen in neutrophils is accompanied by an autoantigen that is defined as a complementary antigen [26]. The complementary antigen molecularly mimics a Staphylococcus aureus antigen. Thus, antibodies against Staphylococcus can cross-react with the complementary antigen, which explains the activation of this disease following pharyngeal infections. An anti-idiotype response to a complementary PR3 (cPR3) protein can also develop [5].

Antibodies against endothelial cells have also been described in ANCA-associated vasculitis [27].

Cellular immunity plays an important role in the pathogenesis of ANCA-associated vasculitis. Thus, T cells are involved in the production of renal lesions in ANCA-mediated crescentic glomerulonephritis by directly participating in crescent formation [28]. A deficiency in the functionality of T-regulatory cells has been identified [29]; however, the role of B cells is less well known. However, favorable results with rituximab treatment also point toward a role for B cells [30].

Dying neutrophils release chromatin fibers that trap extracellular invading microbes, which are called neutrophil extracellular traps (NETs) [31].

NETs were also found in ANCA-associated vasculitis [32]. In the kidneys, anti-MPO-ANCAs can induce the formation of NETs from primed neutrophils. MPO is present in NETs [33]. A NET-associated autoantigen has also been attributed a role in initiating ANCA production [34]. In patients with anti-MPO-ANCA-associated microscopic polyangiitis, the induction of NETs correlated with ANCA affinity for myeloperoxidase and disease activity [35]. Anti-LAMP-2 may also be involved in NET formation [36]. Therefore, NETs could be a novel antigen source [37].

Genetic and epigenetic factors can also intervene in vasculitis [38,39,40]. Microbial organisms, especially Staphylococcus aureus, could be of foremost importance amongst environmental factors [41].

ANCA-associated vasculitis represents the most important cause of crescentic glomerulonephritis; however, further studies are required to elucidate the immune mechanisms involved in its pathogenesis.

**Figure 1:**
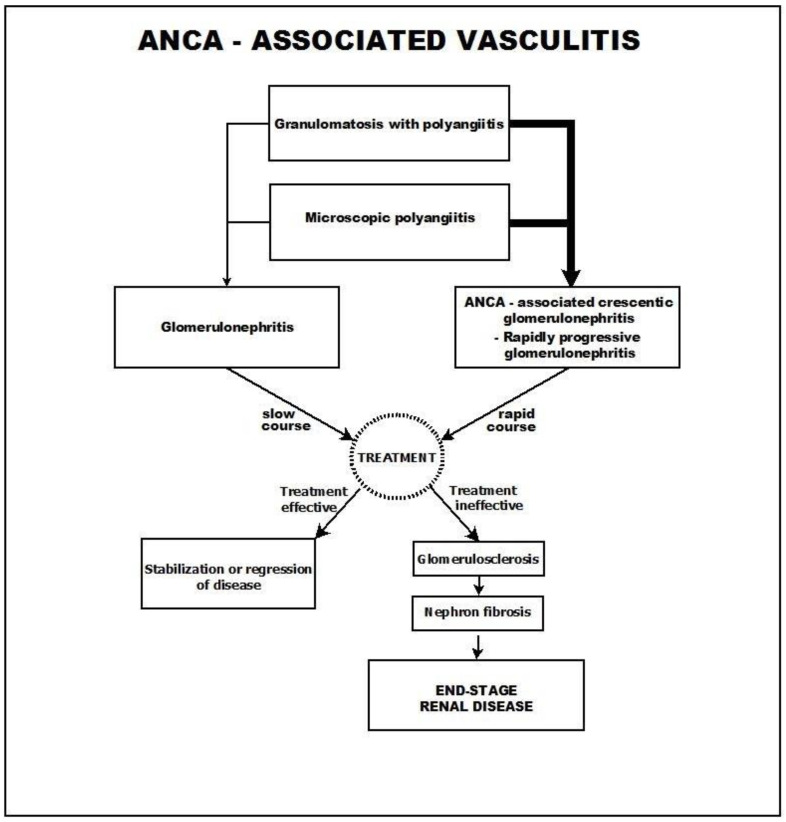
ANCA-associated vasculitis.

An overview of ANCA-associated vasculitis is presented in Figure 1.

## 3. Anti-Glomerular Basement Membrane (GBM) Disease

Anti-glomerular basement membrane (GBM) disease or anti-GBM glomerulonephritis is characterized by the presence of autoantibodies directed against type IV collagen (specifically, the noncollagenous region of its alpha 3 chain) in the GBM. Some cases are associated with antibodies directed against the basement membrane of lung alveoli, producing Goodpasture’s syndrome [42].

Anti-GBM disease can present as rapidly progressive crescentic glomerulonephritis. The disease accounts for approximately 15% of US patients with rapidly progressive, crescentic GN in >50% of glomeruli [2]. These antibodies can also act against the tubular basement membrane (BM); therefore, they are responsible for tubulo-interstitial inflammation. Goodpasture’s syndrome associates not only with rapidly progressive crescentic glomerulonephritis but also with pulmonary hemorrhage.

Genetic factors together with environmental factors, such as hydrocarbons, trichlorethane, carbon tetrachloride, xylene, ethylether, exposure to tobacco smoke, and infection, could be involved in an individual’s susceptibility to Goodpasture’s syndrome [43]. There is a strong genetic linkage to HLA-DRB1*1501 and HLA*DRB1*1502 [44], and HLA B7 is associated with disease severity. According to Ooi et al., the DRB1*0701 and the DRB1*0101 genes are protective against the disease [45]. Furthermore, Ooi et al. demonstrated that the HLA-DRB*1501-restricted Goodpasture’s T cell epitope induces GN [46].

Goodpasture’s syndrome has a complex pathogenesis that involves many autoimmune mechanisms. Notably, the GBM does not usually express epitopes that can cause anti-GBM antibody production. It presents as a hexamer that contains alpha 3, alpha 4, and alpha 5 chains [47]. They contain two protomers with triple-helix structures, and a NC1 domain that has a non-collagenic structure connects them to each other.

The protomers contain two types of epitopes, E (A) and E (B). They are sequestered within the hexamer. When the structure is intact at the GBM level, it ensures ultrafiltration in the kidneys [48].

Under the influence of external factors, such as infection, solvents, tobacco smoke, or unidentified factors, the hexamer structure can be affected. If the GBM cryptic epitopes are exposed, they cause antibody formation. These antibodies are defined as anti-glomerular basement membrane antibodies and trigger an autoimmune reaction. The formation of these autoantibodies, which causes autoimmune phenomena, is accompanied by an inflammatory reaction [49]. As a result, the modified GBM structure undergoes gap formation at this level.

Fibrin, monocytes, and lymphocytes, mainly T cells, pass through these gaps into the filtration area, which results in a reaction of the cells limiting this area, namely the parietal epithelial cells of Bowman’s capsule and podocytes. If they are not removed, these cells will remain in the filtration area, leading to crescents set in superposed layers. Important vascular lesions have recently shifted the classification of Goodpasture’s syndrome to a type of vasculitis [44].

Macrophages and T cells are present in the interstitium. As a result of their actions, Bowman’s capsule lesions occur and form gaps through which these cells can reach the filtration area and contribute to crescent formation. Fibroblasts also reach this area via these gaps.

At the same time, important proliferative processes take place in the glomerulus, and the glomerular function is strongly impaired.

During an unfavorable disease course, fibrosis processes occur at the crescent level. As a consequence, severe rapidly progressive glomerulonephritis occurs, which, without effective therapeutic intervention, can advance to end-stage renal disease.

Red blood cells and proteins can pass through gaps at the GBM level and thus patients present with proteinuria and hematuria.

Anti-GBM antibodies also affect the hexamers at the alveolar BM level, which produce lesions that allow for extravasation of red blood cells into the sputum and, less frequently, pulmonary hemorrhage.

Histologic studies have shown that IgG and, less frequently, IgA deposits are located linearly along the GBM. Linear deposits are also present at the tubular BM level. At the GBM level, areas of fibrinoid necrosis have sometimes been identified.

Electron microscopy can detect gaps in the GBM in areas with focal necrosis. Neutrophils and monocytes are present at this level, and fibrin is present in the filtration area. In cases associated with anti-MPO antibodies, vascular lesions of necrotizing inflammation in arteries or arterioles have been identified. Sometimes, electron microscopy can identify dense electron deposits corresponding to immune complexes. In such cases, Goodpasture’s syndrome is considered to be associated with an immune complex disease, for example, post-streptococcal GN.

Some cases of Goodpasture’s syndrome present without pulmonary involvement. This is known as “renal limited anti-GBM disease” [50].

Normal people regulate immunity to the alpha 3, 4, and 5 NC1 subunits well. When the conformational structure changes, unrestricted antigens are displayed. Then, Goodpasture’s syndrome occurs and is defined as a conformational disease, namely as a conformeropathy [51].

Antibodies against linear epitopes were detected in the serum of these patients. P14 is such an epitope that reacts with T and B cells, and it could have a nephritogenic role [52].

Reynolds et al. pointed to the presence of an antigen complementary to the Goodpasture antigen against which there appeared to be anti-complementarity alpha 3 antibodies in the serum of patients with acute disease. This suggests a “role for autoantigen complementarity in the immunopathogenesis of this disease.” The authors also identified antigen mimicry between microbial antigens and the Goodpasture antigen [53].

Cellular immunity and humoral immunity are considered to have independent pathogenetic nephritogenic roles in anti-GBM nephritis [54].

Anti-GBM antibodies present in the serum of patients with Goodpasture’s syndrome correlated with disease activity, severity, and progression [55].

A pathogenetic role of anti-GBM antibodies is supported by experimental studies. Glomerulonephritis has been induced in sheep following immunization with GBM [56]. Couser considers anti-GBM-mediated disease a prototype autoimmune disease in humans [57].

Mechanisms of cellular immunity also are involved in Goodpasture’s syndrome, the main role being attributed to T cells.

T cells can influence B cell function and antibody production. According to Ooi et al., they have an important pathogenetic role in both the initiation and the subsequent course of Goodpasture’s syndrome [45].

Th1 and Th17 cytokines in the serum increase concomitantly with the development of necrotizing crescentic GN, which could point to their role in producing the disease [58].

Hopfer et al. conducted experimental studies that suggested that autoreactive T cells are able to induce the formation of pathologic autoantibodies [59].

T cells from patients with Goodpasture’s syndrome recognize epitopes in areas that are highly susceptible to auto-antigen processing by autosomal proteases. Reactive oxygen species play an important role in exposing cryptic epitopes [60]. In some experimental studies, Wu et al. suggested the presence of T cells in Bowman’s spaces. They considered that T cells could target antigens in or adjacent to Bowman’s capsules [61]. T cells produce gaps at the level of Bowman’s capsule, which are involved in crescent formation because they pass from the interstitium into the filtration area.

Antigen-specific T cells have been detected in the circulation, and their action has been prevented by α3 (IV) NC1- specific T reg cells [62].

Some patients present both anti-GBM and ANCA antibodies. This double-positive association may be present in 20–30% of patients [63]. The most frequently detected association is anti-GBM antibodies and ANCA anti-myeloperoxidase antibodies. Patients who present with this association display a more severe clinical phenotype and have worse outcomes compared with patients who have only anti-GBM antibodies [64]. Li et al. observed autoantibodies against linear myeloperoxidase epitopes in patients with anti–glomerular basement membrane disease [65]. Serratrice et al. furthered the hypothesis that MPO-ANCA induces anti-GBM antibodies through exposure of cryptic epitopes on alpha 3 (IV) NC1 [66]. Further research studies that provide evidence for the hypothesis of a common pathogenetic mechanism are underway [65]. The histologic findings of double-positive patients differ from those of patients with anti-GBM only [67].

According to Srivastava et al., patients with both anti-GBM and ANCA antibodies have renal manifestations that resemble anti-GBM glomerular disease clinically and histologically, while extra-renal manifestations are similar to those of ANCA-associated vasculitis [68].

**Figure 2:**
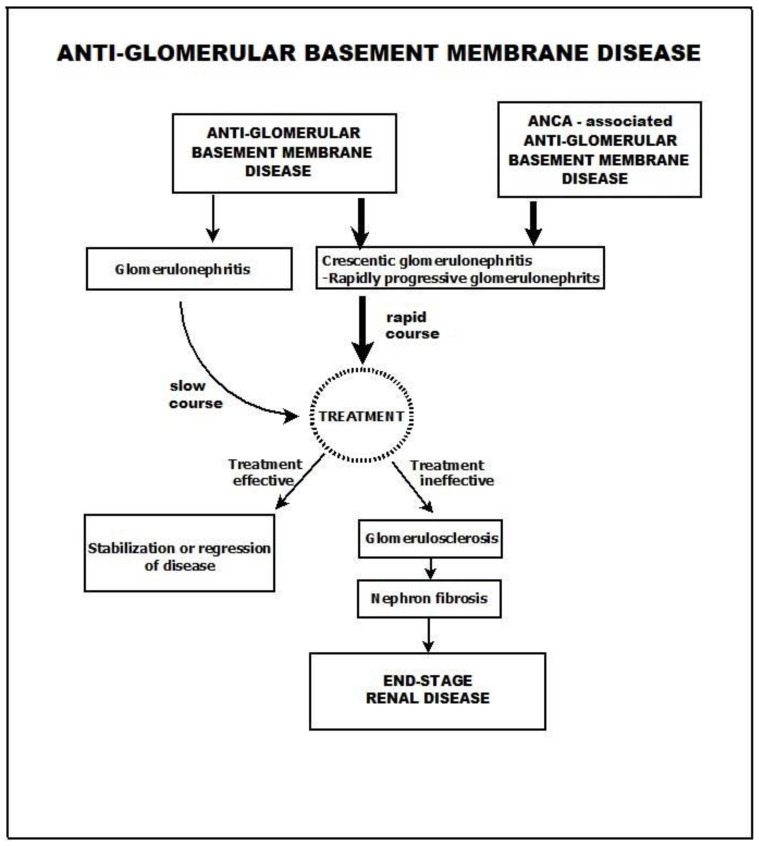
Anti-GBM disease.

An overview of anti-GBM disease is presented in Figure 2.

## 4. Immune Complex-Mediated Crescentic Glomerulonephritis

In this form of crescentic GN, the pathogenetic mechanisms differ from those found in ANCA-associated vasculitis or anti-GBM disease. Immune complex crescentic GN (ICCGN) is mediated by circulating immune complexes or immune complexes formed in situ [69].

It is generally thought that renal lesions produced by immune complexes (IC) are less aggressive and that the RPGN that accompanies them has a more favorable outcome compared with the outcomes of ANCA-associated vasculitis or anti-GBM disease.

ICCGN is found in several glomerular nephropathies that are mediated by IC, the most significant one being observed in lupus nephritis [70]. Another important disease in which nephropathy is mediated by IC is IgA vasculitis, formerly known as Henoch-Schonlein purpura. This disease is more frequently accompanied by crescent formation than other IC-mediated nephropathies [71].

ICCGN diseases comprise the following entities, according to [70,71,72,73,74,75,76,77,78,79,80]; see Table 1.

### 4.1. Crescentic Rapidly Progressive Glomerulonephritis in Lupus Nephritis

SLE is a systemic autoimmune disease, and the kidney is involved in SLE in up to 20–40% of cases [81]. Lupus nephritis is considered to be a “classic immune complex-mediated renal disease” [82], and it is an immune-mediated renal disease with significant renal lesions in the vast majority of patients. Severe lupus nephritis is defined by the widespread (>50%) involvement of glomeruli [83]. Crescentic lupus nephritis represents an extremely severe form of lupus nephritis, which is clinically overt as RPGN.

To better understand lupus nephritis and to define crescentic lesions, the International Society of Nephrology (ISN) developed classifications for the renal lesions in lupus nephritis in 2003.

This classification is based on kidney biopsy histopathology and comprises six classes of lupus nephritis, including the following:

- Class I—minimal mesangial lupus nephritis,
- Class II—mesangial proliferative lupus nephritis,
- Class III—focal lupus nephritis (<50% of glomeruli),
- Class IV—diffuse lupus nephritis (>50% of glomeruli),
- Class V—membranous lupus nephritis, and
- Class VI—advanced sclerosing lupus nephritis.

Class III presents three sub-classes: A—active lesions, AC—associates chronic lesions with active lesions, and C—chronic inactive lesions with scarring.

Class IV encompasses six sub-groups according to the distribution of segmental or global lesions and to activity or inactivity. Class IV comprises active lesions, proliferative lesions, necrotic lesions, and crescent formation [84].

A more recent version of the ISN/Renal Pathology Society (RPS) lupus nephritis classification was published in 2018.

Antibodies against nucleosomes or DNA are formed during lupus nephritis as a consequence of broken tolerance to autoantigens. In SLE, there is decreased ability to degrade DNA [85]. Antibodies against nuclear antigens are present in the circulation in the form of circulating immune complexes, which can be deposited at the kidney level [86]. The localization is related to receptors specific to immunoglobulins (FcR) on the surface of the renal cells to which they attach. At the same time, immune complexes can form in situ [87]. Nuclear antigens have intrinsic affinity for glomerular cells or basement membrane surfaces, and they can play a role as planted antigens for anti-DNA and anti-nucleosome antibodies [88].

The presence of immune complexes in renal structures triggers inflammatory phenomena. Antibodies that form immune complexes are located sub-endothelially along the internal surface of the basement membrane and, less frequently, on the external, sub-epithelial side, presenting as sub-epithelial humps on electron microscopy. At the same time, immune complexes are deposited at the mesangial cell level, where they can induce their proliferation. Membranous lupus nephritis and mesangial proliferative lupus nephritis have been described in these cases [89].

Inflammatory processes accompany the presence of immune complexes at the glomerular basement membrane level. These processes involve the complement system, which eventually leads to formation of membrane attack complexes that produce pores in cell membranes and impair the glomerular basement membrane [90]. The following represents other factors that may be involved:

1. Reactive oxygen species are also produced within the framework of the oxidative stress that accompanies the inflammatory processes triggered by immune complexes and the complement system.
2. Chemokines are released from endothelial cells. Thus, CCL5/RANTES has an important role in recruitment of inflammatory cells to areas of injury [91]. Other chemokines produced by the endothelium may also play a role in lupus nephritis, including fractalkine [92]; interleukin 8, which is an inflammatory cytokine with additional chemokine properties [93]; CXCL10, which is considered a biomarker in lupus patients [94]; and chemoattractant protein-1 [95]. Activation of mesangial cells by nucleosome-containing immune complexes produces chemokines (e.g., CCL2, CCL7, CXCL1, CXCL2, and CXCL7) that have chemotactic roles and produces neutrophil, macrophage, and T and B cell infiltration [96]. The chemokine CXCL13, which is produced during lupus nephritis, has been analyzed as a new SLE and lupus nephritis biomarker [97].
3. T cells can cause disruption of the glomerular basement membrane by means of the granzymes they produce [98].
4. B cells have multiple functions in lupus. One of them is the production of anti-nuclear and polyreactive autoantibodies [88].

The presence of immune complexes can be detected by immunofluorescence techniques, including IgG, IgA, IgM, and complement deposits. These deposits have a granular aspect. Electron microscopy shows electron-dense deposits [99].

Crescent formation in the filtration area is related to fibrin discharge through areas of disruption or holes produced in the glomerular basement membrane. Activation of the Bowman’s capsule parietal epithelial cells as well as podocytes occurs. Macrophages and T cells can penetrate through the impaired areas under the influence of some chemokines. All of these participate in crescent formation. Notably, the nuclear components of immune complexes can play a role in activating macrophages via their toll-like receptors (TLRs) [89]. They worsen the inflammatory processes at this level.

NETs also have an important role in the pathogenicity of this disease [100,101]. In the process of NETosis, neutrophils release extracellular nuclear traps, which can be a local source of nucleosomes [102]. Impaired neutrophil extracellular trap clearance may contribute to immunogenicity in SLE [103].

Periglomerular interstitial inflammatory processes play a lesser role than in vasculitis or in GN produced by anti-GBM antibodies. Thus, as the impairment of Bowman’s capsule is less marked in immune complex-mediated crescentic GN and some macrophages originate in the interstitium, the participation of macrophages in crescent formation is more limited. As a consequence, there is a diminished tendency of crescent fibrosis, and the course of RPGN is usually less severe.

In rare cases, immune complexes are deposited in alveolar capillaries. Capillary lesions with ensuing pulmonary manifestations can occur.

Immune complexes located in glomerular vessels can be associated with necrotizing vascular lesions [104].

In a significant number of patients, ANCAs are also detected [105]. Biopsy examination mainly reveals focal segmental glomerulosclerosis or mesangial proliferative glomerulonephritis lesions [106]. In certain cases, ANCAs may be associated with membranous lupus nephritis [105]. ANCA-associated necrotizing and crescentic glomerulonephritis may present as superimposed nephropathy on lupus nephritis [107]. Patients with crescentic lupus nephritis and ANCAs have a significantly poorer outcome [108].

**Figure 3:**
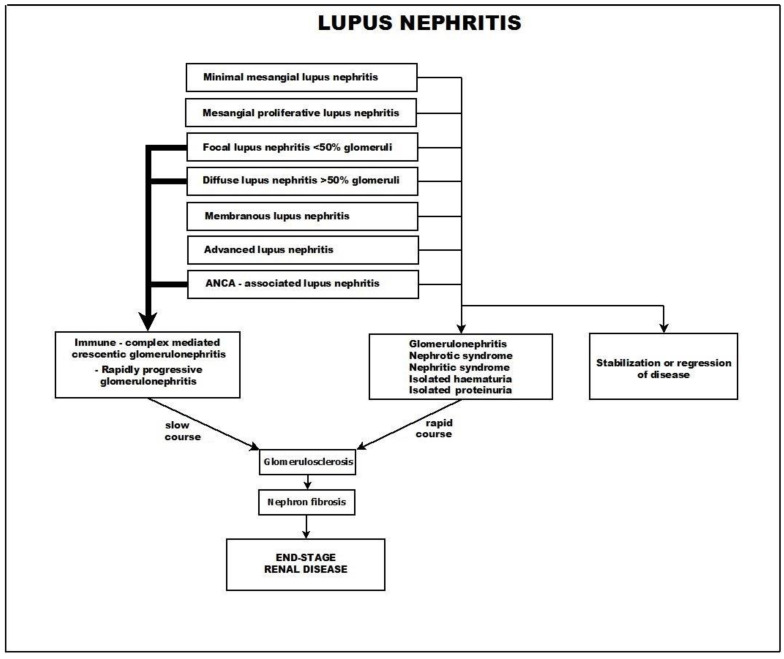
Lupus nephritis.

An overview of lupus nephritis Is presented in Figure 3.

### 4.2. Other Forms of Immune Complex-Mediated Crescentic Glomerulonephritis

Occasionally, other forms of RPGN are observed, for example, during the course of IgA nephropathy. Because of crescent formation, this nephropathy, which is mediated by IgA antibodies, turns into immune complex-mediated crescentic glomerulonephritis with severe outcome. At the glomerular basement membrane level, IgA, C3, sometimes IgG, and, less frequently, IgM deposits may be detected [109].

IgA vasculitis, formerly known as Henoch-Schonlein purpura, sometimes manifests with crescent formation and glomerular IgA deposits. IgA vasculitis is considered to be a part of IgA nephropathy and IgA vasculitis according to the Chapel Hill Conference Classification 2012 [3]. This disease belongs to the vasculitis group, where immune complexes have a pathogenetic role [110].

Post-infectious glomerulonephritis has, in some cases, a severe outcome and renal biopsy reveals crescent formation [75,111,112]. Immunofluorescence shows IgG and other immunoglobulin deposits, as well as C3 in the form of granular deposits.

In membranous nephropathy, C3 and immunoglobulin deposits are formed on the external side of the GBM. Immune complexes formed in situ most commonly mediate membranous nephropathy. Granular IgG deposits are predominant. Membranous nephropathy can be accompanied in rare cases by extra-capillary proliferation, namely by crescents, which leads to a severe disease outcome [76].

Other glomerular nephropathies, such as membranoproliferative glomerulonephritis, can present with RPGN, being associated with crescent formation. Notably, the immune deposits are granular in this nephropathy [77].

An important number of immune complex-mediated glomerular nephropathies with crescents expressed as RPGN are associated with ANCA antibodies. The pathogenetic mechanisms predominant in these cases are immune complex-mediated [78].

## 5. Crescentic Glomerulonepthritis—A Synthesis

The term ‘crescentic glomerulonephritis’ refers to a pathologic condition characterized by extra-capillary proliferation in >50% of glomeruli.

Disruption of the glomerular capillary wall in autoimmune diseases, such as ANCA-associated vasculitis, anti-GBM disease, and immune complex-mediated glomerulonephritis, occurs as a consequence of different pathogenetic mechanisms involving glomerular capillaries [113]. They result in capillary wall disruption with formation of gaps that communicate with the filtration space. Fibrin and leukocytes can pass through these gaps into the filtration area. Inflammatory processes occur and impair the three layers of the capillary wall, including the endothelium, GBM, and visceral epithelial cells (podocytes).

The pathogenesis of crescent formation has been analyzed separately for each of the three autoimmune diseases, with their specific characteristics. In this chapter, we present a brief overview of crescentic glomerulonephritis.

The involved capillaries frequently undergo necrosis, which is most evident in ANCA-associated vasculitis and sometimes in SLE. The necrosis process is usually localized; however, in some cases, such as in ANCA-associated vasculitis, it can involve the whole glomerulus. Fibrin and cells such as macrophages and lymphocytes are the main elements that pass into the filtration area through the disrupted vascular wall [8]. The presence of fibrin in the filtration area produces a cellular proliferative response (parietal epithelial cells (PECs) and podocytes) and induces migration of blood cells (macrophages and T cells) reducing the filtration space. As a consequence, they form layers of sickle-shaped cells, which produce glomerular cellular crescents. Van Roeyen et al. found that overexpression of platelet-derived growth factor-D (PDGF-D) in podocytes induced rapidly progressive crescentic glomerulonephritis and glomerular sclerosis. They considered that cellular crescent formation results from activation of the PDGF-receptor by podocyte-derived PDGF-D [114].

Fibrin passing through capillary gaps facilitates coagulation processes at this level. Coagulation factors are present, and they participate in increased procoagulant activity in the filtration area. Fibrin deposits at the crescent level are related to glomerular tissue factor, which can take part in the coagulation activity at this level [115]. At the same time, the glomerular tissue factor pathway inhibitor activity is diminished, and increased plasminogen activator inhibitor-1 activity may be observed. Factors limiting thrombin formation also have diminished activity [116].

Fibrinolysis processes are also diminished. In fact, thrombi formation processes sometimes occur together with necrosis processes in glomerular capillaries in the vicinity of the filtration area where crescents are formed. They are due both to impairment of the capillary walls and to increased coagulation activity and diminished fibrinolysis.

The importance of the coagulation factors was confirmed in experimental studies on animals, which demonstrated that defibrination with ancrod prevents crescent formation. [117]. Experimental studies have also demonstrated the participation of the plasminogen/plasmin system and that plasmin/plasminogen activators have a protective effect against renal injury in crescentic glomerulonephritis. On the contrary, plasminogen activator inhibitors have an important role in renal injury [118,119].

There is a relationship between the coagulation processes and the other processes that participate in crescent formation. Fibrin can act as a chemotactic factor for macrophages [120].

**Figure 4:**
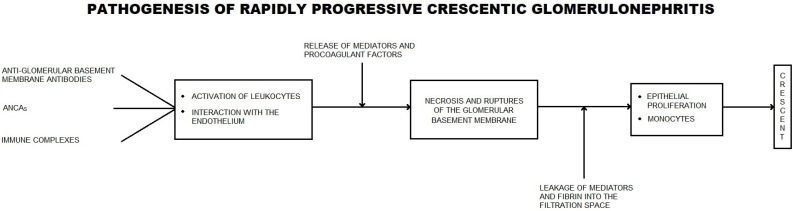
Pathogenesis of rapidly progressive crescentic glomerulonephritis.

An overview of the pathogenesis of rapidly progressive crescentic glomerulonephritis is presented in Figure 4.

### 5.1. Bowman’s Capsule Lesions

In crescentic GN produced by ANCA-associated vasculitis, anti-GBM disease, and immune complex-mediated GN, one of the affected compartments is the tubulo-interstitial compartment. The periglomerular interstitium presents an important inflammatory reaction in which monocyte/macrophage cells are present.

Activated periglomerular mononuclear cells can cause disruption of Bowman’s capsule and produce gaps [9]. Macrophages and activated T cells that become attached to crescents pass through these gaps into the extra-capillary area. In diseases in which lesions of the capsule are absent or limited, such as in immune complex-mediated GN, crescents formed from epithelial cells are predominant. In GN with crescents that impair Bowman’s capsule, macrophages predominate [121]. T cells and macrophages will act chemotactically via the chemokines they produce.

Later, fibroblasts will pass through the gaps, attach to the crescents, and form fibrocellular crescents, which, in time, can cause crescent fibrosis with subsequent renal function impairment.

### 5.2. Parietal Epithelial Cells (PECs)

Plasma leakage can act as a trigger for PEC proliferation and crescent formation [8]. These cells are considered to be well differentiated, with limited potential for complex activity. The PECs associate with macrophage cells, which are known for their phagocytosis capacity, and with T cells. Their presence is related to the activity of some chemotactic factors, including MIP-1 alpha, MIP-1 beta, and MCP-1.

### 5.3. Renal Progenitor Cells

There are several types of progenitors at the kidney level [122]. Renal progenitor cells have an important regenerative role that is exerted on podocytes [123,124].

A subset of renal progenitor cells presents such qualities and includes cells expressing CD24 and CD133 markers [125].

These CD133+ and CD24+ cells are precisely disposed in Bowman’s capsules, being located between the urinary and vascular poles [126].

Renal progenitor cells can migrate via the vascular stalk. When they arrive to a lesional site, they differentiate into podocytes [124]. Cells presenting the CD24+ and CD133+ markers were found at the crescent level [127].

Smeets et al. considered that renal progenitor cells could intervene both in podocytopathies and in crescentic glomerulonephritis [128].

Shankland et al. considered that, in certain circumstances, parietal progenitor cells could augment scarring and crescent formation [123].

### 5.4. Podocytes

Podocytes also participate in crescent formation. They are considered terminally differentiated non-dividing cells [129]. Experimental studies on mice that were conducted in a model of crescentic glomerulonephritis showed that podocytes can form a bridge between the glomerular tuft and Bowman’s capsule and can migrate between parietal cells. Here, they undergo proliferation and become crescents [130]. Podocytes can lose their specific podocyte phenotype when they become crescents [131].

Bariety et al. observed that podocytes that had undergone proliferation and dysregulation could present podocytopathy [132]. According to Wiese et al., podocytes could even form integral cellular components of crescents in glomerular disease [129].

### 5.5. Macrophages

Macrophages have been shown to participate in both intra-capillary and extra-capillary fibrin deposition and crescent formation, as well as to mediate capillary wall damage [57].

Macrophage infiltration is prominent in the acute inflammatory lesions in RPGN and is associated with renal dysfunction and glomerular and tubulo-interstitial histologic damage. Infiltrating macrophages produce a variety of pro-inflammatory factors in these acute lesions, including TNF-alpha, IL-1, nitric oxide synthase (NOS)-2, and macrophage migration inhibitory factor, indicating a classical M1-type macrophage phenotype. However, as acute inflammation is replaced by progressive fibrosis, the role of macrophages in chronic kidney disease becomes less clear. Furthermore, there is an increasing awareness of the heterogeneous nature of macrophage responses and how this is determined by their local microenvironment. Experimental studies of macrophage function in RPGN have focused mainly on models of nephrotoxic serum nephritis (NTN) and lupus nephritis. A variety of strategies to block macrophage infiltration or activation during the development of inflammatory lesions has proven effective in preventing/halting active crescent formation and renal dysfunction, demonstrating that macrophages cause acute renal injury in this setting. In contrast, the role of macrophages in established crescentic disease when predominant inflammatory lesions give way to a progressive fibrotic phase is poorly understood. Thus, it is unclear whether macrophages are a suitable target during the progressive fibrotic phase of RPGN. Studies of macrophages in progressive interstitial fibrosis in the unilateral ureteric obstruction model have produced conflicting results, and these data cannot readily be extrapolated to a model of RPGN. Han et al. have provided evidence that macrophages also contribute to renal dysfunction and tissue damage in established crescentic glomerulonephritis as it progresses from the acute inflammatory to a chronic fibrotic phase [133].

Macrophages come mainly from the circulation, passing into the filtration area through gaps in capillary walls. Several chemokines and osteopontin play important chemotactic roles. Macrophages become attached by means of ICAM-1 and VCAM-1 adhesion molecules.

Macrophages also play an important role in attracting T cells. Once attached at the crescent level, macrophages produce several cytokines, including IL-1, TGF-beta, TNF-alpha, and tissue factor.

Macrophages can express diverse proteinases and matrix metalloproteinases whereby they can disrupt Bowman’s capsule.

At the same time, macrophages intervene in fibrosis processes by means of TGF-beta [133].

### 5.6. Dendritic Cells

Kidney dendritic cells regulate nephritogenic T cells responses [134]. It has been postulated that in lupus nephritis, a subset of CD11+ dendritic cells with a pro-inflammatory role may be present, promoting crescentic glomerulonephritis, and a subset of CD103+ dendritic cells antagonizing this subset and exerting protective effects against crescentic glomerulonephritis may also be involved. This effect is due, according to Evers, to intrarenal accumulation of FoxP3+ T reg cells [134].

### 5.7. T Cells

T cells can intervene in inflammatory processes and affect glomerular capillaries, contributing to their disruption, and leading to gap formation.

T cells can act directly via the granzymes they produce or indirectly by macrophage stimulation with proteolytic enzyme production and by the release of free oxygen species [135].

The presence of T cells in the filtration area is influenced by chemotactic factors produced by monocytes/macrophages. Cytokines (e.g., IL-12 and IL-18) are also involved and induce local inflammation and leukocyte recruitment [136]. Odobasic et al. observed that glomerular expression of CD80 and CD86 could be involved in leukocyte accumulation and injury in crescentic glomerulonephritis [137]. T cells and macrophages are involved in Bowman’s capsule lesions.

Kitching et al. suggested that human crescentic GN could represent a nephritogenic response mediated by Th1 cells, namely delayed-type hypersensitivity (DTH)-like responses [136].

Th17 cells that have a pathogenetic role could play a role in crescentic GN [138]. Th17 cells produce a pro-inflammatory cytokine, which acts by means of the IL-17 receptor (IL-17 RA). In mice, it has been shown that IL-17 RA signaling significantly contributes to renal tissue injury in experimental autoimmune glomerulonephritis and blocking IL-17 RA may be a promising therapeutic strategy for the treatment of proliferative and crescentic glomerulonephritis [139]. On the contrary, IL-10 could have a suppressive role in Th17 cell differentiation [140].

T cells are considered to play an important role in producing autoimmune crescentic GN [141]. According to Chen, JX, and Chen, N, T cells, as well as dendritic cells and toll-like receptors, are involved in podocyte activation and PEC proliferation. Thus, they could participate in producing glomerular injury and crescent formation [142].

### 5.8. Neutrophils

Neutrophils contain a number of serine proteases in their cytoplasmic granules, including cathepsin G, neutrophil elastase, and proteinase 3 (PR3) [143]. These proteases are released at the inflammatory site [144]. They have roles in regulating inflammation and innate immune responses [145].

According to Mihara et al., neutrophil proteinases released at inflammatory sites can affect tissue function by either activating or inhibiting signal transduction mediated by proteinase-activated receptors (PARs). PAR1 is expressed at sites where abundant neutrophil infiltration occurs. Neutrophil-derived enzymes might also regulate PAR signaling, including both PARs 1 and 2, which are present on vascular endothelial cells [143]. Their serine proteases and extracellular nucleosomes enhance tissue factor- and factor XII-dependent coagulation in a process involving local proteolysis of the coagulation suppressor tissue factor pathway inhibitor [146]. FcγR, plays an important role in mediating innate immune responses. FcγRIIB is an important modulator of inflammatory effector cells such as mast cells, neutrophils, and macrophages during the efferent phase of an immune response [147].

Neutrophils can form NETs that can produce cytotoxic effects on endothelial cells, podocytes, and PECs [148]. Once released, proteolytic enzymes can be concentrated in NETs [146]. NET dysregulation could be involved in autoimmune disease pathogenesis [149]. Nakazawa et al. signaled the possible involvement of NETs in the pathogenesis of MPO-ANCA-associated vasculitis [149]. According to Grayson and Kaplan, the elucidation of the subcellular events of NET formation could represent a novel strategy to address the innate immune system in autoimmune and vascular diseases [103].

### 5.9. Fibroblasts

Fibroblasts at the interstitial level undergo proliferation during disease. They penetrate disruption areas, namely gaps in Bowman’s capsule, into the filtration area. They arrive in crescents and attach to them, playing a role in their formation.

TGF-beta can intervene in fibroblast proliferation [120], and their conversion into myofibroblasts [150]. Fibroblast growth factor limits thrombin formation [120].

Epithelial–myofibroblast conversion occurs at the crescent level. Myofibroblasts play an important role in the progression of crescentic glomerulonephritis [150].

During the progression of crescentic glomerulonephritis, a transition from cellular crescents to fibrocellular, and thereafter, fibrous crescents occur [151].

## 6. Course and Outcome

The outcome of untreated rapidly progressive crescentic GN is severe. To date, therapies have had favorable effects, usually halting disease progression. According to Atkins et al., the course is unfavorable in cases of capsular rupture with a predominance of macrophages and fibroblasts in the extra-capillary space [120].

The way in which crescent formation influences the course of crescentic GN is not clear. According to Atkins et al., the progression of crescentic GN can present in two stages, including a stage of active inflammation, which can often be followed by a second stage that consists of fibrocellular and fibrous crescent formation [120].

Kriz et al. observed that although crescent formation could be a consequence of capillary wall impairment with crescent formation, crescents may occlude the outlet from Bowman’s capsule to the proximal tubule. By producing atubular glomeruli, they could intervene in the progression of kidney lesions [152].

Crescents can progress, usually when therapy is absent or inefficient, toward fibrosis. Fibrosis can also be present at the level of other glomerular structures.

At the same time, the clinical picture of crescentic GN has an unfavorable outcome.

The severity of the disease is related to the nature and type of immunologic process causing the disease and in part to the degree of crescent formation. Patients with circumferential crescents in more than 80 percent of the glomeruli tend to present with advanced kidney failure that may not respond well to therapy. Patients with fewer, non-circumferential crescents tend to have a more indolent disease course.

Notably, not all of the cases of the above-mentioned diseases associate during their crescentic GN disease progression renal function impairment [153]. 

**Figure 5:**
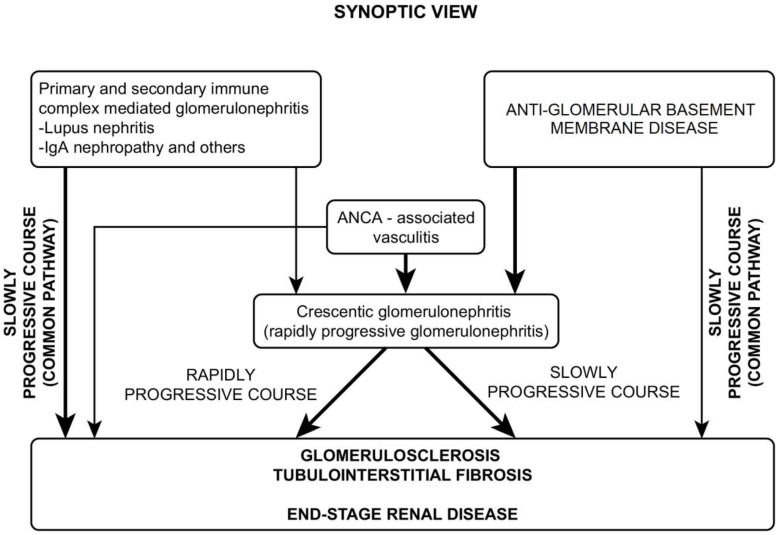
Synoptic view.

Two pathways of progression of glomerular diseases can be delineated: the first is a common slowly progressive pathway, which is most frequently encountered, and the second pathway is rapidly progressive glomerulonephritis, which presents with the most common histologic features of crescentic glomerulonephritis. The progression pathways are depicted in Figure 5.

Without therapy, RPGN progresses to ESRD over a period of days to weeks to a few months. Patients with fewer, non-circumferential crescents may have a more protracted, not so rapidly progressive course.

The initial therapy of most patients with RPGN involves pulse methylprednisolone followed by daily oral prednisone, oral or intravenous cyclophosphamide or rituximab, and, in some cases, plasma exchange. Appropriate therapy is essential to minimize the degree of irreversible kidney injury. The results of therapy are favorable in many patients and halt the progression of rapidly progressive glomerulonephritis, which untreated can rapidly progress toward ESRD. It is to be noted that three autoimmune diseases with different pathogenesis benefit from a similar immunosuppressive or immunomodulating treatment. Jennette defined this therapy as standard treatment [2]. Progress in the therapy of crescentic glomerulonephritis includes anti-TNF-alpha agents, complement antagonists, anti-IL-6 receptor monoclonal antibody, tyrosine kinase inhibitors, and antibody immunoadsorption) [154].

## 7. Conclusions

The three different immune mechanisms involved in glomerular nephropathies can have in common crescent formation at the glomerular level. Clinically, they are forms of rapidly progressive glomerulonephritis.

We suggest common expression mechanisms of severe autoimmune diseases that impair the kidney. The immune mechanisms in ANCA-associated vasculitis, anti-GBM disease, and immune complex-mediated GN, although different, have a common histopathologic and clinical presentation—rapidly progressive crescentic glomerulonephritis.

Immune-mediated renal diseases can take two different courses to terminal stages:

- A common course, in which the glomerulosclerosis process advances slowly and progressively and is usually accompanied by tubulo-interstitial fibrosis.
- A different course, in which the presenting picture rapidly progresses toward crescentic glomerulonephritis.

The pathogenetic treatment aimed at the immune processes in these diseases is similar and involves corticosteroids, immunosuppressive or immunomodulating drugs, and sometimes plasma exchange. Therapy should take into consideration the specifics of each disease.

## Figures and Tables

**Table 1:** Causes of immune complex-mediated crescentic glomerulonephritis.

	IMMUNE COMPLEX-MEDIATED CRESCENTIC GLOMERULONEPHRITIS
1.	Lupus nephritis
2.	IgA nephropathy
3.	IgA vasculitis
4.	Hepatitis B and C secondary glomerulonephritis
5.	Post-infectious glomerulonephritis
6.	Membranous nephropathy
7.	Membranoproliferative glomerulonephritis
8.	Fibrillary nephropathy
9.	Cryoglobulinemia glomerulonephritis
10.	Abscesses
11.	Idiopathic

## Data Availability

Not applicable.
